# Integrating Heat Training in the Rehabilitation Toolbox for the Injured Athlete

**DOI:** 10.3389/fphys.2019.01488

**Published:** 2019-12-11

**Authors:** Mohammed Ihsan, Julien D. Périard, Sébastien Racinais

**Affiliations:** ^1^Aspetar Orthopaedic and Sports Medicine Hospital, Doha, Qatar; ^2^Research Institute for Sport and Exercise, University of Canberra, Canberra, ACT, Australia

**Keywords:** heat acclimation, heat therapy, physiotherapy (rehabilitation), muscle hypertrophy, muscle atrophy, passive heating, thermal therapy

## Introduction

Musculoskeletal injuries are arguably one of the most severe impediments athletes may encounter in their career. Depending on the severity of the sustained injury, time-loss could be substantial, leading to profound de-conditioning effects within the cardiovascular, metabolic and muscular systems. For instance, following ACL reconstruction, athletes' return to sport may range between 16 and 52 weeks (Anderson et al., 2019). Case studies in elite soccer players have reported profound losses in whole body fat-free mass (5.8 kg), as well as in lean leg mass (0.9–1.5 kg) following immobilization and inactivity during this period (Milsom et al., 2014; Anderson et al., 2019). Moreover, a 4–20% decline in maximal aerobic capacity (VO_2max_) has been reported following 2–8 weeks of physical de-conditioning, owing largely to the decline in blood volume, and consequently stroke volume and cardiac output (Mujika and Padilla, 2001). In this context, there is a crucial need to optimize rehabilitation programs, such that the physical demands associated with return to sport and beyond are well-tolerated.

**Figure 1:**
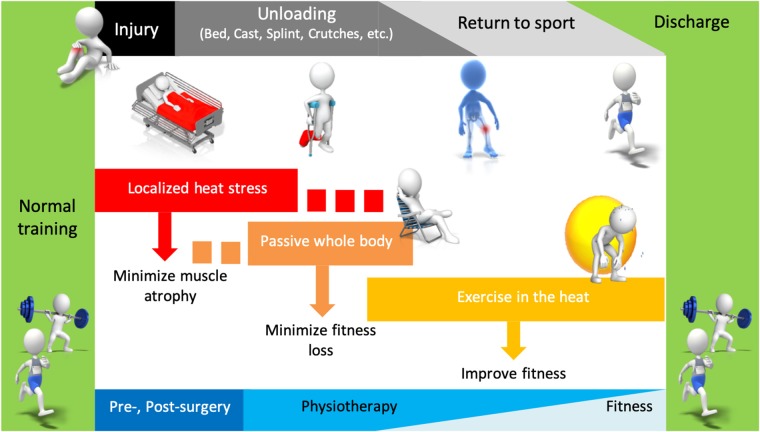
Depiction of how the therapeutic use of passive or active heat stress may be utilized at different stages of rehabilitation to limit muscle atrophy and decline in fitness, optimizing the return to sport.

Here we propose that an athlete's rehabilitation program may be optimized by incorporating repeated heat exposures or heat acclimation, given the evidence in support of this modality to minimize skeletal muscle and cardiovascular de-conditioning during periods of disuse and reduced physical activity. For example, there is emerging evidence demonstrating remarkable benefits of heat stress on the regulation of muscle mass (Selsby and Dodd, 2005; Selsby et al., 2007; Ihsan et al., 2014; Hafen et al., 2019). Moreover, training in the heat allows for maintaining a lower absolute work load for a given relative exercise intensity, reducing the mechanical demands of the rehabilitating athlete. While it is important to note that some (Shvartz et al., 1977; Sawka et al., 1985; Lorenzo et al., 2010), but not all (Karlsen et al., 2015; Keiser et al., 2015; Neal et al., 2016) studies have reported improved exercise performance in cooler environments following heat acclimation, harnessing the benefits through passive or active heat acclimation remains a promising strategy to minimize cardiovascular de-conditioning in a rehabilitating athlete, whose training load is considerably reduced. This opinion piece presents how repeated heat exposures may be incorporated in the rehabilitation toolbox at various stages of the return to sport journey (Figure 1).

## Muscle Adaptations to Heat Therapy to Minimize Muscle Atrophy When Immobilized

During the first phase of the rehabilitation program, including pre- and post-surgery, heat exposure may be used to minimize the loss in muscle mass (Figure 1). Indeed, the effects of heat stress on the regulation of muscle mass has been relatively well-studied in cell cultures and rodents, demonstrating remarkable benefits. For instance, heat stress has been shown to enhance the recovery of muscle mass within atrophied muscles (Goto et al., 2004; Selsby et al., 2007), as well as attenuate the loss in muscle mass in aging (Ohno et al., 2012), disuse (Selsby and Dodd, 2005), and pharmacological (Tsuchida et al., 2017) models of muscle atrophy. Moreover, heat exposure has been shown to preserve muscle mass and minimize protein degradation in a variety of muscle trauma models including ischemic injury (Garramone et al., 1994), crush injury (Takeuchi et al., 2014), exercise-induced muscle damage (Touchberry et al., 2012), and pharmacologically-induced muscle toxicity (Kojima et al., 2007; Shibaguchi et al., 2016).

The potential for heat-based interventions to be an integrative part of a rehabilitation strategy has, therefore, gathered considerable enthusiasm lately. This surge in interest is in-part due to recent studies in humans demonstrating improved muscle contractile function (Racinais et al., 2017b) or attenuated loss of muscle mass during immobilization (Hafen et al., 2019) following short-term heat exposure. Specifically, Racinais et al. (2017b) showed that 11 days of passive whole body heat exposure (60 min @ 48–50°C, 50% RH) resulted in an increased peak twitch amplitude, improved maximal voluntary torque, as well as the relative torque/electromyographic relationship. Additionally, more recent data by Hafen et al. (2019) showed that the decline in muscle mass following 10 days of lower limb immobilization was in-part attenuated when 120 min of localized heating of the vastus lateralis was administered daily. The preserved muscle mass following heat treatment coincided with the protein abundance of the transcriptional co-activator PGC-1α, HSP 70, and HSP 90, as well as minimized loss of mitochondrial respiratory chain protein content and mitochondrial respiratory function (Hafen et al., 2019). These findings are in general agreement with evidence gathered from cultured cells and rodents, where heat stress had been shown to mitigate muscle atrophic pathways through HSPs, PGC-1α, and mitochondrial signaling (Sandri et al., 2006; Romanello et al., 2010; Cannavino et al., 2015).

In addition to a mitochondrial and HSP centered mechanism, data gathered from cell cultures and rodent models implicate the Akt-mTOR cascade as a potential pathway by which heat stress might preserve muscle mass during immobilization. For instance, acute exposures (30–60 min) to environmental heat (39–41°C) has been shown to increase anabolic signaling (i.e., Akt-mTOR pathway), in line with increased muscle protein content (Goto et al., 2003; Uehara et al., 2004; Ohno et al., 2012; Yoshihara et al., 2013). Apart from initiating protein synthesis *per se*, activation of Akt has also been shown to suppress protein degradation through inactivating FOXO3, a key transcription factor regulating the expression of atrophic genes (Sandri et al., 2004). Nevertheless, while there is considerable evidence indicating that Akt-mTOR signaling may be up-regulated following heat stress, further evidence involving humans is further needed to verify this pathway within immobilization and injury models.

## Passive Heat Acclimation to Minimize De-conditioning Effects When Not Training

In addition to protecting muscle mass, passive heat exposures may also help to maintain cardiovascular fitness from the onset of injury (Figure 1). Indeed, the cardiovascular and thermoregulatory adaptations conferred by repeated heat exposures are somewhat similar to the adaptations acquired through exercise *per se*, but with some specificities to improve thermoregulation. Importantly, a large part of the cardiovascular and thermoregulatory adaptations conferred by exercise heat stress may also be obtained through passive exposures in the condition that they sufficiently trigger an increase in body temperature, circulation, and sweating (Racinais et al., 2015; Périard et al., 2016). With recent studies demonstrating the accrue of such adaptations following passive heat exposures (Brazaitis and Skurvydas, 2010; Racinais et al., 2017a,b), there is intuitively an interest in developing heat-related therapeutic modalities for injured athletes restricted from training. The purpose of such modalities would be to induce cardiovascular adaptations (e.g., blood volume expansion) that aid in attenuating the loss of fitness, given the deterioration in cardiovascular adaptation substantially accounts for the decrease in VO_2max_ during de-conditioning (Mujika and Padilla, 2001). Practical passive heat acclimation strategies include hot water immersion or sauna bathing. Saunas are typically 80–90°C, whereas water temperature when immersing into a bath should be 40–42°C to induce adaptation, while remaining tolerable. These modalities are typically undertaken for 30–60 min, depending on whether heating was preceded by exercise. Guidelines regarding passive heating strategies are comparable to exercise heat acclimation and include consecutive days of exposure with a minimum of 5–7 exposures for initiating adaptations (Heathcote et al., 2018).

## Heat Training to Optimize the Relative/Absolute Workload in Injured Athletes

Once the athlete can progressively load the injured limb but is still unable to undertake heavy training, heat exposure during mild exercise may enhance the re-conditioning process (Figure 1). Indeed, exercise heat exposure provides a unique challenge to the cardiovascular system, which must not only supply blood to exercising muscles (i.e., oxygen delivery), but also to the peripheral vasculature (i.e., heat loss). When prolonged exercise is undertaken under heat stress and whole-body temperature increases, the cardiovascular response is progressively exacerbated compared to that of temperate conditions (Périard et al., 2013). This exacerbation is characterized by a compromise in the maintenance of mean arterial blood pressure and cardiac output, as heart rate drifts upward and stroke volume decreases (Rowell, 1974; Coyle and Gonzalez-Alonso, 2001). This forces the cardiovascular system toward a functional limit (i.e., VO_2max_) (Nybo et al., 2001; Arngrimsson et al., 2003) with a progressive hyperthermia-induced reduction in VO_2max_ increasing the relative exercise intensity (i.e., %VO_2max_) for a given absolute workload (Périard et al., 2011; Périard and Racinais, 2015). Thus, the hyperthermia-induced dissociation between relative and absolute exercise intensity (Périard and Racinais, 2015) may allow injured athletes to train at a given heart rate or percentage of VO_2max_ for a lower absolute mechanical load in hot than temperate conditions.

Although the exacerbated cardiovascular response inherent with exercising under heat stress impairs the ability to perform optimally in hot environments, it may provide a pathway for injured athletes to train at high relative intensities without having to produce high levels of mechanical work with an injured limb. For example, injured athletes could exercise at a workload equivalent to a particular %VO_2max_, which as the session progressed, would result in increasing cardiovascular (Wingo, 2015) and metabolic (Febbraio et al., 1994) responses. To expedite these responses and avoid having to produce high levels of mechanical work in the early part of exercise sessions, prior to the development of whole-body hyperthermia, exercise may be preceded by passive heating.

Ultimately, we suggest that injured athletes may benefit from training in the heat as the cardio-metabolic load associated with a given mechanical workload increases progressively with the development of hyperthermia. This may help maintain fitness or attenuate the loss of aerobic capacity associated with a prolonged rehabilitation process following an injury, particularly in those unable to fully load the injured limb.

## Conclusion

In summary, the therapeutic use of either exercise or passive heat exposure is a novel and emerging modality to assist with post-injury rehabilitation or re-conditioning. The use of such modalities can be targeted toward minimizing cardiovascular de-conditioning in athletes who are unable to undertake their usual mechanical loading. Moreover, drawing from the extensive research in rodent and cell cultures, as well as emerging evidence in humans, the use of passive heat application may minimize the decline in muscle mass during immobilization or periods of severely restricted exercise. There is a need though, to undertake further research toward optimizing the delivery of passive and active heat treatment modalities (i.e., whole-body vs. limb only, optimal muscle temperature and duration, dose-response, etc.). Such information would aid in the development of evidence-based heat therapy protocols for both sporting and clinical situations.

## Author Contributions

MI, JP and SR conceived and contributed to writing the manuscript. All authors provided critical feedback approved the final version of the manuscript.

### Conflict of Interest

The authors declare that the research was conducted in the absence of any commercial or financial relationships that could be construed as a potential conflict of interest.
